# Energy valley optimizer: a novel metaheuristic algorithm for global and engineering optimization

**DOI:** 10.1038/s41598-022-27344-y

**Published:** 2023-01-05

**Authors:** Mahdi Azizi, Uwe Aickelin, Hadi A. Khorshidi, Milad Baghalzadeh Shishehgarkhaneh

**Affiliations:** 1 Department of Civil Engineering, University of Tabriz, Tabriz, Iran; 2 School of Computing and Information Systems, The University of Melbourne, Melbourne, Australia; 3 Department of Civil Engineering, Islamic Azad University of Tabriz, Tabriz, Iran

**Keywords:** Computer science, Computational science

## Abstract

In this paper, Energy Valley Optimizer (EVO) is proposed as a novel metaheuristic algorithm inspired by advanced physics principles regarding stability and different modes of particle decay. Twenty unconstrained mathematical test functions are utilized in different dimensions to evaluate the proposed algorithm's performance. For statistical purposes, 100 independent optimization runs are conducted to determine the statistical measurements, including the mean, standard deviation, and the required number of objective function evaluations, by considering a predefined stopping criterion. Some well-known statistical analyses are also used for comparative purposes, including the Kolmogorov–Smirnov, Wilcoxon, and Kruskal–Wallis analysis. Besides, the latest Competitions on Evolutionary Computation (CEC), regarding real-world optimization, are also considered for comparing the results of the EVO to the most successful state-of-the-art algorithms. The results demonstrate that the proposed algorithm can provide competitive and outstanding results in dealing with complex benchmarks and real-world problems.

## Introduction

In today's competitive world, many efforts are allocated to maximize the overall benefits of nature's limited resources. Recent advances in human knowledge and expertise reveal the need to consider greater accuracy, better performance, and higher construction speeds in the design of real-world systems. It is necessary to develop new methods for design and optimization and implement them on real complex problems to achieve each of the mentioned factors. Optimization is a decision-making process in which the search for a function's minimum and/or maximum values ​​is considered. Optimization algorithms are divided into two categories as exact and approximate algorithms. Exact algorithms can find the optimal solution accurately. Still, in the case of complex optimization problems, they are not efficient enough, and their computational time increases exponentially according to the dimensions of the problem. So, given the limitations of exact methods and the need for precision and speed in identifying appropriate answers, approximate algorithms, like metaheuristics, can find suitable solutions close to the optimal solution in a shorter time that can be used to solve complex problems. In fact, the Greek prefix “meta”, shown within the title, is utilized to demonstrate that these algorithms are “higher-level” heuristic algorithms differentiating with problem-specific heuristics^1^. The metaphors used by scholars while developing new metaheuristics are as follows: natural evolution, insects, gravitation, electromagnetic force, ecosystem, water, plant, human, birds, and animals. Among them, “insects” is the most often used metaphor for simulating social behaviour to develop practical optimization problems, and bees are the most frequently used insect metaphor, followed by ants^2^. Although metaheuristic algorithms give reasonable results, they do not provide optimum solutions.

Generally speaking, there remain four major classifications of metaheuristics based on the source of their inspiration. The first group is evolutionary algorithms (EAs), robust and flexible metaheuristic search algorithms based on Darwinian evolution that efficiently capture global solutions to complicated optimization problems. EAs replicate the biological course of evolution at the cellular level to create better candidate solutions using selection, crossover, mutation, and reproduction operators. The genetic algorithm (GA) introduced by John Holland and his colleagues in 1992 could be deemed as the so-called EA^3^. Another prominent algorithm is differential evolution (DE) which was introduced in 1997^4^. In 2014, another evolutionary-based metaheuristic algorithm called hyper-spherical search (HSS) was proposed^5^. The algorithm's core principle was searching the hypersphere's inner space, which is defined by the hypersphere's core and its particles. Swarm-based metaheuristic algorithms are the second category. Particle swarm optimization (PSO) is a well-known and often utilized swarm intelligence algorithm. The basic concept of PSO was inspired by a swarm of flying birds looking for food^6^. Furthermore, in 2012, another swarm-based algorithm named firefly algorithm (FA) was introduced^7^. As its name implies, this algorithm was inspired by the flashing behaviour of fireflies. Ant colony optimization (ACO) is another swarm-based algorithm that uses ants' foraging behaviour. ACO solves a problem by maintaining an updated pheromone trail and relocating these ants in the search space using simple mathematical calculations based on the area's transition probability and total pheromone^8,9^. Among the other well-known algorithms in this category are rat swarm optimizer (RSO)^10^; the wisdom of artificial crowds (WoAC)^11^; tuna swarm optimization (TSO)^12^; and artificial bee colony (ABC)^13^.

The Big-Bang Big-Crunch (BBBC) algorithm is inspired by the theories of the universe's evolution. Another prominent algorithm is henry gas solubility optimization (HGSO), imitating the behavior governed by Henry's law^14^. Moreover, Atomic Orbital Search (AOS) is another algorithm in which the fundamental principles of quantum physics are used as a source of inspiration^15^. Several more well-known algorithms in this categorization include the following: Material Generation Algorithm (MGA)^16,17^, cyber-physical systems (CPS)^18^, Archimedes optimization algorithm (AOA)^19^, Lichtenberg Algorithm (LA)^20^, and also Thermal Exchange Optimization algorithm (TEOA)^21^. Finally, the fourth group is human and animal lifestyles-based metaheuristic algorithms. Harris Hawks Optimizer (HHO) is one of the renowned animal behaviour-based algorithms; the cooperative behaviour and pursuit manner of Harris' hawks in nature, known as surprise pounce, is the fundamental inspiration for HHO^22^. In terms of efficiency, quality of results, and acceptable convergence in dealing with various applications in real-world problems, the HHO has gotten much attention from academics^23^. An artificial Jellyfish Search (JS) optimizer has recently been proposed in 2021, inspired by the behaviour of jellyfish in the ocean^24^. Additionally, queuing search (QS) is another algorithm based on human queueing behaviors^25^. Table 1 contains more information on the algorithms mentioned above. Additionally, there are other metaheuristic optimization algorithms inspired by different concepts which have been proposed in the last years, including the following: cooperation search algorithm (CSA)^26^, Aquila Optimizer (AO)^27^, Capuchin Search Algorithm (CapSA)^28^, Golden Tortoise Beetle Optimizer (GTBO)^29^, Battle Royale Optimization (BRO)^30^, passing vehicle search (PVS)^31^, Dynamic Virtual Bats Algorithm (DVBA)^32^, crow search algorithm (CSA)^33^, virus optimization algorithm (VOA)^34^, bald eagle search (BES) algorithm^35^, Gravitational Search Algorithm (GSA)^36^, Grey Wolf Optimizer (GWO)^37^, Teaching–Learning-Based Optimization (TLBO)^38^, Fire Hawk Optimizer (FHO)^39,40^, Social Spider Optimization (SSO)^41^, League Championship Algorithm (LCA)^42^, and Chaos Game Optimization^43,44^.

**Table 1:** Historical to cutting-edge metaheuristic optimization algorithms.

Name	Authors	Year	Classifications	Inspiration
GA^3^	J Holland	1992	Evolutionary-based	Charles Darwin's theory of natural evolution
Wisdom of Artificial Crowds (WoAC)^11^	R Yampolskiy et al	2012	Swarm-based	Human collective intelligence
Bat algorithm (BA)^45^	X Yang et al	2012	Nature-based	The echolocation behaviour of bats
Hyper-Spherical Search (HSS) algorithm^5^	H. Karami et al	2014	Evolutionary-based	Space search mechanism
Passing vehicle search (PVS)^31^	P Savsani et al	2016	Population-based	Vehicle passing on a two-lane highway
Dynamic Virtual Bats Algorithm (DVBA)^32^	A Topal et al	2016	Nature-based	Bat's echolocation behaviour
Crow search algorithm (CSA)^33^	A Askarzadeh	2016	Population-based	The intelligent behaviour of crows
Virus optimization algorithm (VOA)^34^	Y Liang et al	2016	Population-based	The behaviour of viruses attacking a living cell
Thermal Exchange Optimization algorithm^21^	A Kaveh et al	2017	Physics-based	Newton's law of cooling
Queuing search (QS)^25^	J Zhang et al	2018	Human-based	Human activities in queuing
Henry gas solubility optimization (HGSO)^14^	F Hashim et al	2019	Physics-based	The behaviour governed by Henry's law
Bear smell search algorithm (BSSA)^46^	A Ghasemi-Marzbali	2020	Nature-based	The dynamic behaviours of bear
Bald eagle search (BES) algorithm^35^	H. A. Alsattar et al	2020	Nature-based	The hunting strategy or intelligent social behaviour of bald eagles
Black Widow Optimization Algorithm (BWO)^47^	V Hayyolalam et al	2020	Nature-based	The unique mating behaviour of black widow spiders
Jellyfish Search (JS) optimizer ^24^	J Chou et al	2021	Animal-based	The behaviour of jellyfish in the ocean
Cooperation search algorithm (CSA)^26^	Z Feng et al	2021	Population-based	The team cooperation behaviours in modern enterprise
Aquila Optimizer (AO)^27^	L Abualigah et al	2021	Population-based	The Aquila's natural habits when catching prey
Capuchin Search Algorithm (CapSA)^28^	M Braik et al	2021	Nature-based	The dynamic behaviour of capuchin monkeys
Artificial lizard search optimization (ALSO)^48^	N Kumar et al	2021	Animal-based	The dynamic foraging behaviour of Agama lizards
Archimedes optimisation algorithm (AOA)^19^	F Hashim et al	2021	Physics-based	Law of physics Archimedes' Principle
Rat Swarm Optimizer (RSO)^10^	G Dhiman et al	2021	Bio-based	The chasing and attacking behaviours of rats
Golden Tortoise Beetle Optimizer (GTBO)^29^	O Tarkhaneh et al	2021	Nature-based	The golden tortoise beetle's color-changing habit of attracting the opposite sex
Battle Royale Optimization (BRO)^30^	T Rahkar Farshi	2021	Population-based	A genre of digital games knowns as "battle royale
Lichtenberg Algorithm (LA)^20^	J Pereira et al	2021	Physics-based	The Lichtenberg figures patterns

In recent decades, one of the main challenges of artificial intelligence experts has been the applicability of the proposed metaheuristic algorithm in different fields to optimize and improve the overall efficiency of some specific problems. Artificial electric field algorithm for engineering design optimization^49^, black widow algorithm for engineering optimization^47^, earthquake engineering optimization of structures^50^, engineering design optimization with queuing search algorithm^25^, cuckoo search algorithm for optimization of the travelling salesman problem^51^, optimum design of engineering problems with sine cosine grey wolf optimizer^52^, engineering design optimization with self-adaptive Rao algorithm^53^, design optimization of numerical and engineering optimization problems with improved Harris Hawks optimizer^54^, unconstrained and constrained optimization by hybrid pathfinder optimizer^55^, improved charged system search for optimization of fuzzy controllers^56^, optimum design of structural systems with metaheuristics^57,58^ are some of the most recent research works in the area of applied artificial intelligence.

Even though there are several metaheuristic algorithms, more are always needed. The No Free Lunch (NFL) theory holds that no one method can be used to solve all optimization problems. Thus, the introduction of novel metaheuristic optimization algorithms is continuously ongoing. The creation of new metaheuristics is advantageous to science since they might improve the precision or effectiveness of the optimization procedure for a host of problems^59^. This assertion drives our attempts to suggest a unique metaheuristic algorithm inspired by the cutting-edge physics concepts regarding stability and various forms of decay in particles. Meanwhile, accurate algorithms ensure the most optimal solution, but the problem is that these algorithms are difficult problems. They do not work quickly, and the time to find solutions to complex problems will increase exponentially, and for the hardest and most complex problems, the results of the exact algorarehm are not satisfactory. The approximate algorithms should be utilized if the optimal response to the exact algorithm is not achievable in practice. The approximate or metaheuristic algorithms seek the right solution and are close to optimal. This method lowers the calculation time compared to the previous method.

In this paper, Energy Valley Optimizer (EVO) is proposed as a novel metaheuristic algorithm inspired by advanced physics principles regarding stability and different modes of particle decay. Since the basic principles of the decay process through different particles in physics are used as the main idea of the EVO, the originality of this study may be seen from an inspirational standpoint, while the complexity level of the test functions used is also being examined for the first time in this study. The performance of different algorithms must be done in the same conditions and under the same problems, and under various examples, the superiority of each algorithm cannot be confirmed or denied. Hence, 20 unconstrained mathematical test functions are utilized in different dimensions to evaluate the proposed algorithm's performance. For statistical purposes, 100 independent optimization runs are conducted for determining the statistical measurements as the mean, standard deviation, and the required number of objective function evaluations. A predefined stopping criterion is also considered based on a maximum number of 150,000 objective function evaluations and a tolerance of $1\times {10}^{-12}$ for the global best values of the considered problems. Some well-known statistical analyses, including the Kolmogorov–Smirnov, Wilcoxon, Mann–Whitney, Kruskal–Wallis, and Post-Hoc analysis, are also utilized for comparative purposes. One of the most significant shortcomings of the newly developed metaheuristic algorithms is the simplicity of the evaluation test functions; therefore, two of the latest Competitions on Evolutionary Computation (CEC), the CEC 2020 on bound constraint optimization^60^ and CEC 2020 on real-world optimization^61^, and also the “Big O notation” are considered for comparing the results of the EVO to the most successful state-of-the-art algorithms. However, being parameter-free, fast convergence behaviour, and the lowest possible objective function evaluation could be deemed the privileges of the EVO. In stark contrast, the EVO cannot provide exact solutions; in other words, like other metaheuristic algorithms, the EVO is an approximate algorithm.

Since the proposed EVO is an algorithm developed based on some general and advanced principles of physics, the most important aspect of this algorithm is the conformity between the concept and the mathematical model for which the EVO has higher levels of adaptation between these two aspects. The second factor is the complexity of the algorithm while three new position vectors are created in the main search loop so the recent development in computer science regarding software and hardware allow experts to create algorithms with higher levels of complexity. The third factor is the dynamic configuration of the main loop of EVO in which the exploration and exploitation procedures are conducted by searching the variables’ and candidate’s spaces simultaneously to reach the global best solution. Furthermore, despite the fact that there are several metaheuristic algorithms, more are always needed. Besides, the introduction of novel metaheuristic optimization algorithms is continuously ongoing. The creation of new metaheuristics is advantageous to science since they might improve the precision or effectiveness of the optimization procedure for a host of problems. This assertion drives our attempts to suggest a unique metaheuristic algorithm inspired by the cutting-edge physics concepts regarding particles’ stability and decay.

The main contributions of this study are as follows:

- Advanced physics concepts concerning stability and various forms of decay in particles are examined and analyzed to develop a mathematical model of a metaheuristic algorithm.
- A unique physics-inspired algorithm as Energy Valley Optimizer (EVO) is developed using the mentioned model.
- The EVO's solution updating is dependent on the particles’ enrichment bound, position vector, and stability level.
- EVO's performance is extensively evaluated against a set of twenty benchmark functions and real-world engineering design problems.
- The proposed EVO is compared to a plethora of cutting-edge metaheuristic algorithms.

The rest of the paper is divided into the following sections. In Sect. 2, the inspiration and mathematical model of the proposed EVO are presented. The numerical investigations, including 20 of the best-known mathematical test functions and statistical analysis, including the Kolmogorov–Smirnov, Wilcoxon, and Kruskal–Wallis analysis are indicated in Sects. 3, and 4 respectively. The CEC 2020 complexity analysis and Big O notation are represented in Sect. 5 while the CEC 2020 real-world constrained optimization problems such as Speed Reducer, Hydro-Static Thrust Bearing, Ten-Bar Truss, and also Rolling Element Bearing are elucidated in Sect. 6. Finally, in Sect. 7, the core findings of this study are presented as concluding remarks.

## Energy valley optimizer

### Inspiration

**Figure 1:**
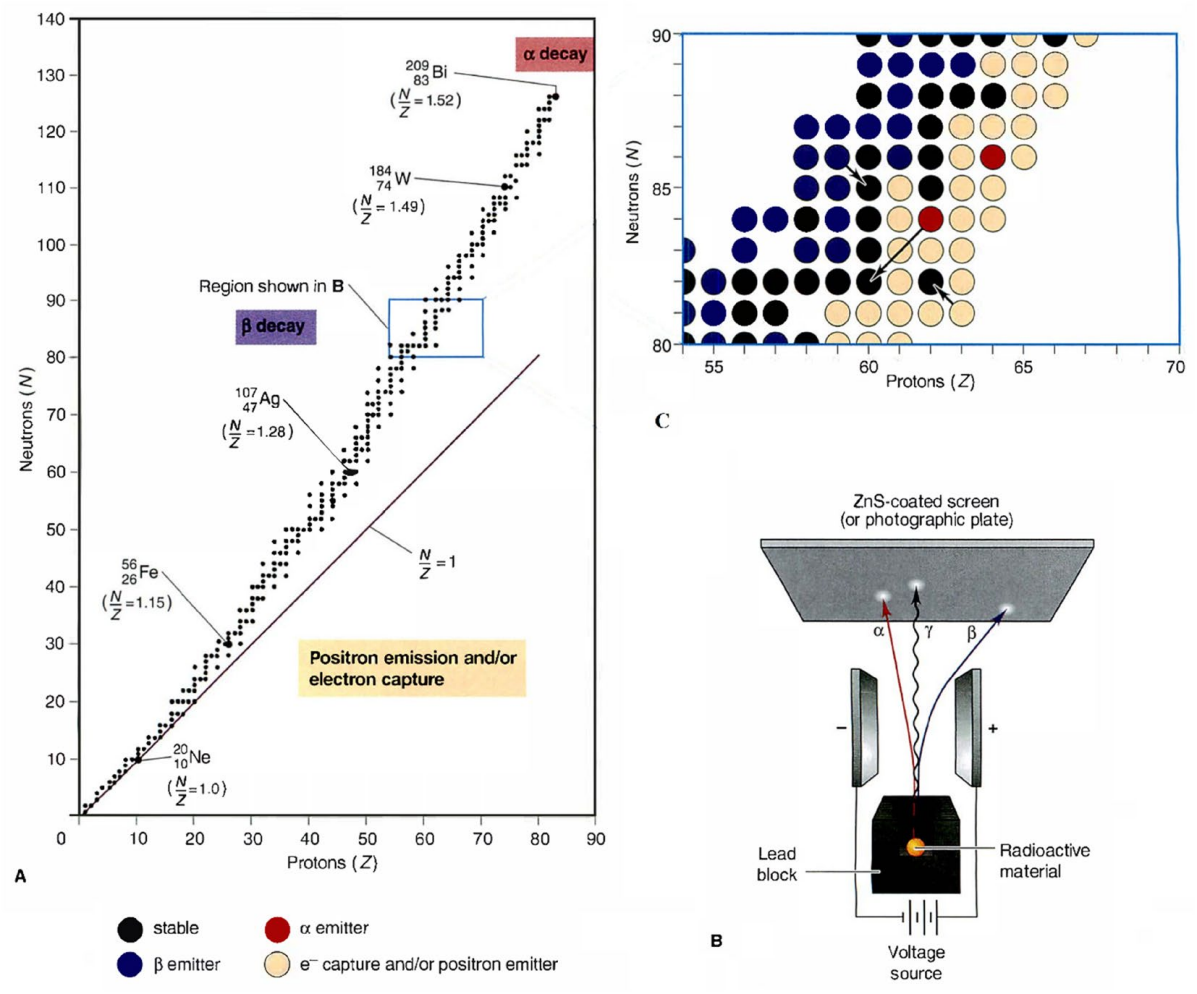
(**A**) Stability band for particles (**B**) Process of emission (**C**) Different types of decay^62^.

Physical reaction refers to colliding two particles or external subatomic particles to produce new particles. In the universe, a great majority of particles are assumed to be unstable except for the stable ones which remain intact indefinitely. The unstable particles tend to emit energy through disintegration or decay, while the overall decay rate is somehow different in various types of particles. In the decaying process, a particle with lower energy is generated while the extra energy is bring-off through the emission process. Energy valley concerns the stability of particles based on their binding energy and interactions with other particles. The direct observation of multiple phenomena has led the experts to extract some valuable patterns for defining the decay in particles. The most crucial challenge in this area is determining the particles’ stability bound by considering the number of neutrons (N) and protons (Z) and the N/Z ratio. The N/Z ≈ 1 refers to the stable, lightweight particle, while for heavier ones, a larger value for N/Z is considered as the stability band. Based on the stability level of the particles, each particle tends to increase its stability level by shifting its N/Z ratio and moving toward the stability band or energy valley. In this regard, the neutron enrichment levels of particles play an essential role in this action. The neutron-rich particles positioned above the stability bound undergo a decay process and require so many neutrons for stability purposes. On the other hand, the neutron-poor particles, which require too few neutrons for stability purposes, tend to undergo electron capture or positron emission to move toward the energy valley or stability band^62^; in Fig. 1.A, these aspects are illustrated schematically.

In the decay process, a particle with a lower energy level is generated while excessive energy is emitted. There are three types of emissions that determine the decay process in particles with different levels of stability. The alpha (α) particles denote the dense and positively charged particles identical to the helium. The beta (β) particles are negatively charged particles characterized as electrons with higher speeds. The gamma (γ) rays represent photons with higher levels of energy. In Fig. 1B, the overall behaviour of these types of emissions is illustrated inside the electric field, considering the α particles curve toward the negative plate to a small extent. In contrast, the β particles curve toward the positive plate greatly, and the electric field does not affect the γ rays.

Based on the presented details of the emission process, there are three types of decay, known as alpha, beta, and gamma decay derived from the previously mentioned emission types. In the alpha decay, the loss of an α particle is determined in which the N and Z values in the N/Z ratio are reduced per emission process. In beta decay, the ejection of a β particle is a concern in which the N/Z ratio is increased by reducing the N and increasing the Z values. In gamma decay, omitting a γ photon with higher energy levels from an excited particle is concerned, while no change in N/Z values is necessary for this mode of decay. The schematic presentation of these aspects is presented in Fig. 1C.

Most of the recently developed metaheuristic algorithms are some reasonable higher-level searching techniques formulated by miscellaneous inspirational concepts to provide one or numerous good solutions for a maximization or minimization optimization problem, particularly with imperfect or incomplete information. The searching processes are primarily conducted among some initial candidates determined randomly. The higher-level procedures of the metaheuristics try to improve the overall standing of these candidates in a step-by-step manner. Hence, the principles of the decay process through different particles can be a great starting point for a novel algorithm in which particles' tendency to achieve a stable point can be utilized as the inspirational concept for performance improvement of the solution candidates.

### Mathematical model

In this section, the EVO is presented as an optimization algorithm in detail by means of the previously described principles of physics. In the first step, the initialization process is conducted in which the solutions candidates (${\mathrm{X}}_{\mathrm{i}}$) are assumed to be particles with different levels of stability in the search space, which is assumed to be a specific part of the universe.

1 $$\mathrm{X}=\left[\begin{array}{c}{\mathrm{X}}_{1}\\ {\mathrm{X}}_{2}\\ \vdots \\ {\mathrm{X}}_{\mathrm{i}}\\ \vdots \\ {\mathrm{X}}_{\mathrm{n}}\end{array}\right]=\left[\begin{array}{c}{\mathrm{x}}_{1}^{1} {\mathrm{x}}_{1}^{2} \cdots {\mathrm{x}}_{1}^{\mathrm{j}} \cdots {\mathrm{x}}_{1}^{\mathrm{d}}\\ {\mathrm{x}}_{2}^{1} {\mathrm{x}}_{2}^{2} \cdots {\mathrm{x}}_{2}^{\mathrm{j}} \cdots {\mathrm{x}}_{2}^{\mathrm{d}}\\ \vdots \vdots \vdots \ddots \vdots \\ {\mathrm{x}}_{\mathrm{i}}^{1} {\mathrm{x}}_{\mathrm{i}}^{2} \cdots {\mathrm{x}}_{\mathrm{i}}^{\mathrm{j}} \cdots {\mathrm{x}}_{\mathrm{i}}^{\mathrm{d}}\\ \vdots \vdots \vdots \ddots \vdots \\ {\mathrm{x}}_{\mathrm{n}}^{1} {\mathrm{x}}_{\mathrm{n}}^{2} \cdots {\mathrm{x}}_{\mathrm{n}}^{\mathrm{j}} \cdots {\mathrm{x}}_{\mathrm{n}}^{\mathrm{d}}\end{array}\right], \left\{\begin{array}{c}\begin{array}{c}i=\mathrm{1,2},\dots ,n.\end{array}\\ j=\mathrm{1,2},\dots ,d.\end{array}\right.$$

2 $${\text{x}}_{{\text{i}}}^{{\text{j}}} = {\text{x}}_{{{\text{i}},{\text{min}}}}^{{\text{j}}} + {\text{rand}}.\left( {{\text{x}}_{{{\text{i}},{\text{max}}}}^{{\text{j}}} - {\text{x}}_{{{\text{i}},{\text{min}}}}^{{\text{j}}} } \right),{ }\quad \left\{ {\begin{array}{*{20}c} {\begin{array}{*{20}c} {i = 1,2, \ldots ,n.} \\ \end{array} } \\ {j = 1,2, \ldots ,d.} \\ \end{array} } \right.$$

where $\mathrm{n}$ denotes on the total number of particles (solution candidates) in the universe (search space); $\mathrm{d}$ is the dimension of the considered problem; ${\mathrm{x}}_{\mathrm{i}}^{\mathrm{j}}$ is the *jth* decision variable for determining the initial position of the *ith* candidate; ${\mathrm{x}}_{\mathrm{i},\mathrm{min}}^{\mathrm{j}}$ and ${\mathrm{x}}_{\mathrm{i},\mathrm{max}}^{\mathrm{j}}$ represent the lower and upper bounds of the *jth* variable in the *ith* candidate; $\mathrm{rand}$ is a uniformly distributed random number in the range of [0, 1].

In the second step of the algorithm, the Enrichment Bound (EB) for the particles is determined, which is utilized for considering the differences between the neutron-rich and neutron-poor particles. For this purpose, the objective function evaluation for each of the particles is conducted and determined as the Neutron Enrichment Level (NEL) of the particles. The mathematical presentation of these aspects is as follows:

3 $${\text{EB}} = \frac{{\mathop \sum \nolimits_{{{\text{i}} = 1}}^{{\text{n}}} {\text{NEL}}_{{\text{i}}} }}{{\text{n}}},{\text{ i}} = 1,2, \ldots ,{\text{n}}.{ }$$

where ${\text{NEL}}^{{\text{i}}}$ is the neutron enrichment level of the *ith* particle, and ${\text{EB}}$ is the enrichment bound of the particles in the universe.

In the third step, the stability levels of the particles are determined as follows based on the objective function evaluations:

4 $${\text{SL}}_{{\text{i}}} = \frac{{{\text{NEL}}_{{\text{i}}} - {\text{BS}}}}{{{\text{WS}} - {\text{BS}}}},{\text{ i}} = 1,2, \ldots ,{\text{n}}.{ }$$

where ${\mathrm{SL}}^{\mathrm{i}}$ is the stability level of the *ith* particle, $\mathrm{BS}$ and $\mathrm{WS}$ are the particles with best and worst stability levels inside the universe equivalent to the minimum and maximum values of so far found objective function values.

**Figure 2:**
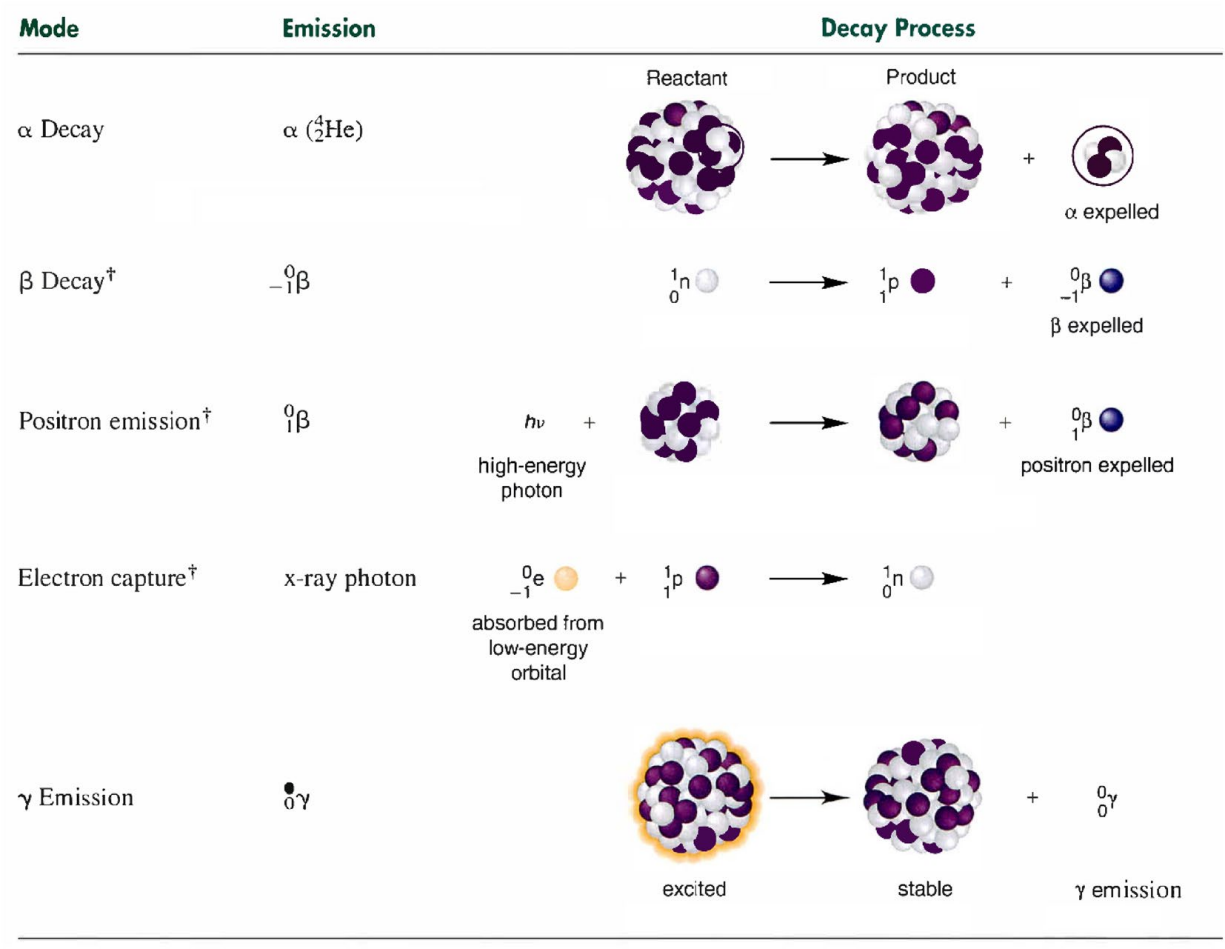
Different modes of decay^62^.

In the main search loop of the EVO, if the neutron enrichment level of a particle is higher than the enrichment bound (${\mathrm{NEL}}_{\mathrm{i}}>\mathrm{EB}$), the particle is assumed to have a larger N/Z ratio, so the decay process utilizing alpha, beta, or gamma schemes are in perspective. In this regard, a random number is generated in the range of [0, 1], which mimics the Stability Bound (SB) in the universe. If the stability level of a particle is higher than the stability bound (${\mathrm{SL}}_{\mathrm{i}}>\mathrm{SB}$), the alpha and gamma decay is considered to happen since these two decays are probable for heavier particles with higher stability levels. Based on physics principles regarding alpha decay (Fig. 2), α rays are emitted to improve the product's stability level in the physical reaction. This aspect can be mathematically formulated as one of the position updating schemes of the EVO in which a new solution candidate is generated. For this purpose, two random integers are generated as Alpha Index I in the range of [1, d], which denotes the number of emitted rays, and Alpha Index II in the range of [1, Alpha Index I], which defines which α rays to be emitted. The emitted rays are decision variables in the solution candidate, which are removed and substituted by the rays in particle or candidate with the best stability level (${\mathrm{X}}_{\mathrm{BS}}$). These aspects are mathematically formulated as follows:

5 $${\text{X}}_{{\text{i}}}^{{{\text{New}}1}} = {\text{X}}_{{\text{i}}} { }\left( {{\text{ X}}_{{{\text{BS}}}} { }\left( {{\text{ x}}_{{\text{i}}}^{{\text{j }}} } \right)} \right),{ }\left\{ {\begin{array}{*{20}c} {\begin{array}{*{20}c} {i = 1,2, \ldots ,n.} \\ \end{array} } \\ {j = Alpha \,Index \,II.} \\ \end{array} } \right.{ }$$

where ${\mathrm{X}}_{\mathrm{i}}^{\mathrm{New}1}$ is the newly generated particle in the universe, ${\mathrm{X}}_{\mathrm{i}}$ is the current position vector of the *ith* particle (solution candidates) in the universe (search space), ${\mathrm{X}}_{\mathrm{BS}}$ is the position vector of the particle with the best stability level, ${\mathrm{x}}_{\mathrm{i}}^{\mathrm{j}}$ is the *jth* decision variable or emitted ray.

Besides, in gamma decay, γ rays are emitted to improve the excited particles' stability level (Fig. 2), so this aspect can be mathematically formulated as another position-updating process of the EVO in which a new solution candidate is generated. For this purpose, two random integers are generated as Gamma Index I in the range of [1, d], which denotes the number of emitted photons, and Gamma Index II in the range of [1, Gamma Index I], which defines which photons to be considered in the particles. The photons in the particles are decision variables in the solution candidate, which are removed and substituted by a neighboring particle or candidate (${\mathrm{X}}_{\mathrm{Ng}}$), which mimics the interaction of the excited particles with other particles or even magnetic fields. In this regard, the total distance between the considered particle and the other ones is calculated as follows, and the nearest particle is utilized for this purpose:

6 $${\text{D}}_{{\text{i}}}^{{\text{k}}} = \sqrt {\left( {{\text{x}}_{2} - {\text{x}}_{1} } \right)^{2} + \left( {{\text{y}}_{2} - {\text{y}}_{1} } \right)^{2} } ,{ }\left\{ {\begin{array}{*{20}c} {i = 1,2, \ldots ,n.} \\ {k = 1,2, \ldots ,n - 1.} \\ \end{array} } \right.$$

where ${\text{D}}_{{\text{i}}}^{{\text{k}}}$ is the total distance between the *ith* particle and the *kth* neighbouring particle, and (${\text{x}}_{1} ,{\text{y}}_{1}$) and (${\text{x}}_{2} ,{\text{y}}_{2}$) denote the coordinates of the particles in the search space.

Using these actions, the position updating process for generating the second solution candidate in this phase is conducted as follows:

7 $${\text{X}}_{{\text{i}}}^{{{\text{New}}2}} = {\text{X}}_{{\text{i}}} { }\left( {{\text{ X}}_{{{\text{Ng}}}} { }\left( {{\text{ x}}_{{\text{i}}}^{{\text{j }}} } \right)} \right),{ }\left\{ {\begin{array}{*{20}c} {\begin{array}{*{20}c} {i = 1,2, \ldots ,n.} \\ \end{array} } \\ {j = Gamma\, Index \,II.} \\ \end{array} } \right.{ }$$

where ${\mathrm{X}}_{\mathrm{i}}^{\mathrm{New}2}$ is the newly generated particle in the universe, ${\mathrm{X}}_{\mathrm{i}}$ is the current position vector of the *ith* particle (solution candidate) in the universe (search space), ${\mathrm{X}}_{\mathrm{Ng}}$ is the position vector of the neighbouring particle around the *ith* particle, and ${\mathrm{x}}_{\mathrm{i}}^{\mathrm{j}}$ is the *jth* decision variable or emitted photon.

If the stability level of a particle is lower than the stability bound (${\mathrm{SL}}_{\mathrm{i}}\le \mathrm{SB}$), beta decay is considered to happen because this type of decay happens in more unstable particles with lower stability levels. Based on the physics principles regarding beta decay (Fig. 2), β rays are expelled from the particles to improve the stability level of the particle, so a big jump in the search space is supposed to be conducted due to the higher levels of instability in these particles. In this regard, a position updating process is conducted for the particles in which a controlled movement toward the particle or candidate with the best stability level (${\mathrm{X}}_{\mathrm{BS}}$) and the centre of particles (${\mathrm{X}}_{\mathrm{CP}}$) is performed. These aspects of the algorithm mimic the particles' tendency to reach the stability band in which most of the known particles are positioned near this band, and most of them have higher levels of stability (Fig. 1a and b). These aspects are mathematically formulated as follows:

8 $${\text{X}}_{{{\text{CP}}}} = \frac{{\mathop \sum \nolimits_{{{\text{i}} = 1}}^{{\text{n}}} {\text{X}}_{{\text{i}}} }}{{\text{n}}},{\text{ i}} = 1,2, \ldots ,{\text{n}}.{ }$$

9 $${\text{X}}_{{\text{i}}}^{{{\text{New}}1}} = {\text{X}}_{{\text{i}}} + \frac{{\left( {{\text{r}}_{1} \times {\text{X}}_{{{\text{BS}}}} - {\text{r}}_{2} \times {\text{X}}_{{{\text{CP}}}} } \right)}}{{{\text{SL}}_{{\text{i}}} }},{\text{ i}} = 1,2, \ldots ,{\text{n}}.{ }$$

where ${\text{X}}_{{\text{i}}}^{{{\text{New}}1}}$ and ${\text{X}}_{{\text{i}}}$ are the upcoming and current position vectors of the *ith* particles (solution candidates) in the universe (search space),${\text{ X}}_{{{\text{BS}}}}$ is the position vector of the particle with the best stability level, ${\text{X}}_{{{\text{CP}}}}$ is the position vector for the centre of particles, ${\text{SL}}^{{\text{i}}}$ is the stability level of the *ith* particle, ${\text{r}}_{1}$ and ${\text{r}}_{2}$ are two random numbers in the range of [0, 1] which determine the amount of particles’ movement.

In order to improve the exploitation and exploration levels of the algorithm, another position updating process is conducted for the particles employing beta decay in which a controlled movement toward the particle or candidate with the best stability level (${\text{X}}_{{{\text{BS}}}}$) and a neighbouring particle or candidate (${\text{X}}_{{{\text{Ng}}}}$) is performed while the stability level of the particle does not affect the movement process. These aspects are mathematically formulated as follows:

10 $${\text{X}}_{{\text{i}}}^{{{\text{New}}2}} = {\text{X}}_{{\text{i}}} + \left( {{\text{r}}_{3} \times {\text{X}}_{{{\text{BS}}}} - {\text{r}}_{4} \times {\text{ X}}_{{{\text{Ng}}}} } \right),{\text{ i}} = 1,2, \ldots ,{\text{n}}.{ }$$

where ${\mathrm{X}}_{\mathrm{i}}^{\mathrm{New}2}$ and ${\mathrm{X}}_{\mathrm{i}}$ are the upcoming and current position vectors of the *ith* particle (solution candidates) in the universe (search space),${\mathrm{ X}}_{\mathrm{BS}}$ is the position vector of the particle with the best stability level, ${\mathrm{X}}_{\mathrm{Ng}}$ is the position vector of the neighbouring particle around the *ith* particle, and ${\mathrm{r}}_{3}$ and ${\mathrm{r}}_{4}$ are two random numbers in the range of [0, 1] which determine the amount of particles’ movement.

If the neutron enrichment level of a particle is lower than the enrichment bound (${\mathrm{NEL}}_{\mathrm{i}}\le \mathrm{EB}$), the particle is assumed to have a smaller N/Z ratio, so the particle tends to undergo electron capture or positron emission to move toward the stability band. In this regard, a random movement in the search space is determined for considering these sorts of movements as follows:

11 $${\text{X}}_{{\text{i}}}^{{{\text{New}}}} = {\text{X}}_{{\text{i}}} + {\text{r}},{\text{ i}} = 1,2, \ldots ,{\text{n}}.{ }$$

where ${\mathrm{X}}_{\mathrm{i}}^{\mathrm{New}}$ and ${\mathrm{X}}_{\mathrm{i}}$ are the upcoming and current position vectors of the *ith* particles (solution candidates) in the universe (search space), and $\mathrm{r}$ is a random number in the range of [0, 1] which determines the amount of particles’ movement.

**Figure 3:**
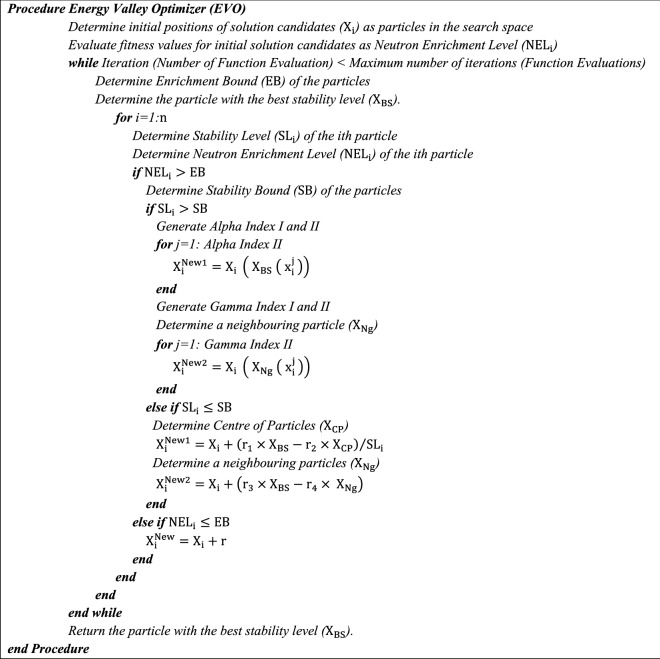
Pseudo-code of the EVO.

**Figure 4:**
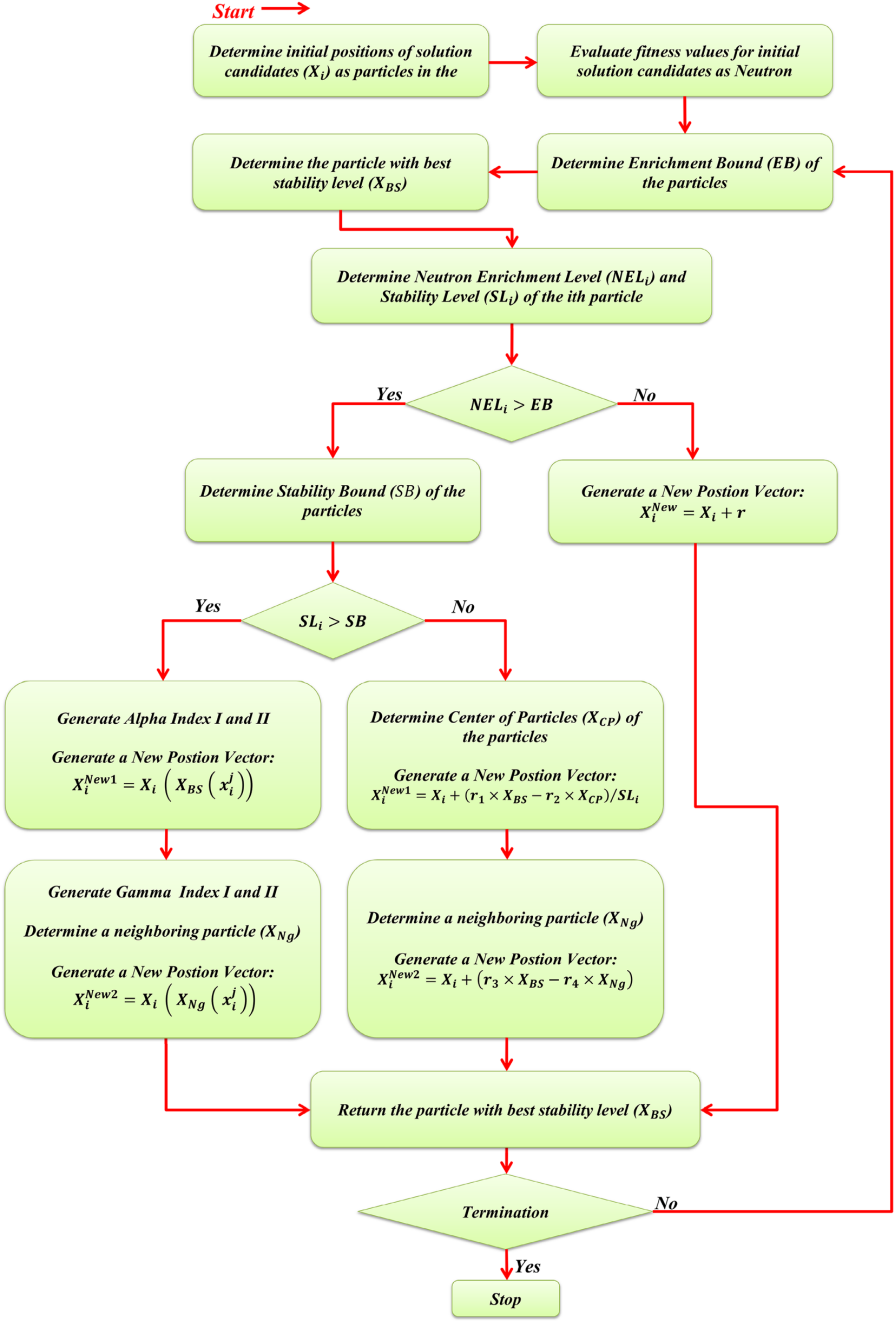
Flowchart of the EVO.

At the end of the main loop of the EVO, there are only two newly generated position vectors for each of the particles as ${\mathrm{X}}_{\mathrm{i}}^{\mathrm{New}1}$ and ${\mathrm{X}}_{\mathrm{i}}^{\mathrm{New}2}$ if the enrichment level of the particle is higher than the enrichment bound, while for the particle with a lower enrichment level, only ${\mathrm{X}}_{\mathrm{i}}^{\mathrm{New}}$ is generated as a new position vector. At each state, the newly generated vectors are merged with the current population, and the best particles participate in the following search loop of the algorithm. A boundary violation flag is determined for the decision variables which go beyond the predefined upper and lower bounds, while a maximum number of objective function evaluations or a maximum number of iterations can be utilized as a termination criterion. The pseudo-code of the EVO is presented in Fig. 3, while the flowchart of this algorithm is provided in Fig. 4.

Overall, one of the primary areas of physics is studying the interactions of particles, which focuses on the unique properties of particles and the elements that make them up as well as their interactions with other particles. While the overall rate of decay varies across different kinds of particles, unstable particles tend to demonstrate emission by disintegration or decay. Determining the bound stability of particles by taking into account the number of neutrons (N) and protons (Z), and determination of the N/Z ratio is the toughest challenge in this field. The neutron-rich particles located above the stability bound, however, experience decay and need many neutrons to be stable. Consequently, the principles of the decay process via various particles can be a fantastic starting point for a unique algorithm in which the solution candidates' performance can be improved by taking inspiration from particles' propensity to reach a stable point.

Considering the new things of this algorithm, the main loop of the algorithm includes three position updating process. Two of these procedures occur in decision variables for which the exploration process is conducted while one position updating process occurs in the solution candidates for which the exploitation is satisfied. The challenging pat of this algorithm is the fact that the exploration part may lead the algorithm to local optimum solutions but the other part tries to tune the previous solutions to reach the global best candidate.

## Mathematical test functions

In this section, 20 of the well-known mathematical test functions are utilized to evaluate the EVO's performance as a novel metaheuristic algorithm. These functions are as Ackley 1, Alpine 1, Chung Reynolds, Exponential, Inverted cosine wave, Pinter, Rastrigin, Salomon, Schwefel 1.2, Schwefel 2.21, Griewank, Powell Singular 2, Schumer Steiglitz, Schwefel 2.4, Schwefel 2.25, Sphere, Step 3, Trigonometric 2, W / Wavy, and Xin-She Yang 6 functions while the first 10 functions are considered with 50 dimensions and the other half are with 100 dimensions. The global best for the fourth, fifth and eighteenth functions are as -1, -49 and 1 respectively while for the rest of them, the global best is as 0. For statistical purposes, 100 independent optimization runs are conducted to determine the statistical measurements as the mean, standard deviation, and the required number of objective function evaluations. A predefined stopping criterion is also considered based on a maximum number of 150,000 objective function evaluations and tolerance of $1\times {10}^{-12}$ for the global best values of the supposed problems.

The parameter settings of the optimization algorithms are shown in Table 2, and all tests to evaluate the EVO's performance were conducted with 50 populations using a PC with the detailed parameters shown in Table 3. In Table 4, the best, mean and Standard Deviation (SD) of results for EVO and other alternative algorithms, including the Ant Colony Optimization (ACO), Harmony Search (HS), Firefly Algorithm (FA), Multiverse Optimizer (MVO), Interior Search Algorithm (ISA), and Cuckoo Search Algorithm (CSA) in dealing with these benchmark mathematical functions are presented. Based on the provided results in these tables, it is obvious that the proposed EVO can outperform the other algorithms in most cases. Considering the required Objective Function Evaluations (OFE), ACO, FA, HS, and CSA required a mean of 150,000 OFE while the MVO with 149,900, ISA with 141,263.90, and EVO with 43,060.28 have better performance. Meanwhile, the same random state in each of the considered algorithms for each optimization run is set to a fixed state to have an unbiased and fair judgment regarding the overall performance of the EVO.

**Table 2:** The parameter settings of the optimization algorithms.

Algorithm	Parameter	Value
ACO	*Q*	1
	*Alpha (α)*	1
	*Beta (β)*	1
	*rho*	0.05
HS	*bw*	0.2
	*HMCR*	0.95
	*PAR*	0.3
FA	*Alpha (α)*	0.25
	*Beta (β)*	0.2
	*Gamma (γ)*	1
MVO	*WEP*_*max*_	1
	*WEP*_*min*_	0.2
ISA	*Alpha (α)*	0.2
CSA	*Pa*	0.25
	*nd*	0.15

**Table 3:** Details of the utilized system in optimization process in the current study.

Name		Detailed settings
Hardware	CPU	CORE i7
	Frequency	2.8 GHz
	RAM	8 GB
	Hard drive	2 TB

**Table 4:** The best, mean and standard deviation of different metaheuristics in dealing with mathematical functions.

No.		Alternative metaheuristic algorithms						
		ACO	FA	HS	MVO	CSA	ISA	EVO
F_1_	Best	20.70461	20.38123	3.655346	0.069087	3.424855	1.41E−05	0
	Mean	20.97241	20.50565	4.372899	3.203908	9.174743	1.56	0.125987
	SD	0.086512	0.050224	0.313316	6.133982	3.58599	4.09E−01	1.25982
F_2_	Best	75.36033	5.423342	0.408871	1.955532	9.844303	7.06E−07	0
	Mean	95.46201	8.546599	0.767617	7.655639	20.88773	4.92E−04	5.47E−13
	SD	6.486329	1.509117	0.150857	3.045156	4.369272	1.16E−03	5.47E−12
F_3_	Best	19,933,773	1.09E + 09	31,448.5	0.006724	0.044259	0	0
	Mean	75,539,630	1.72E + 09	67,983.71	0.024618	6.177559	1.28E−08	0
	SD	34,603,873	3.04E + 08	24,225.86	0.010664	8.326967	9.02E−08	0
F_4_	Best	−0.79993	−0.99974	−0.99117	−1	−1	−1	−1
	Mean	−0.65791	−0.99967	−0.98724	−0.99999	−1	−1	−1
	SD	0.063929	3.13E−05	0.002225	1.65E−06	1.08E−06	5.64E−09	0
F_5_	Best	−4.85263	−13.5479	−39.3724	−13.6182	−15.0676	−22.634	−49
	Mean	−2.99902	−10.0326	−36.6063	−8.73447	−11.5189	−12.5943	−48.5529
	SD	0.411566	1.273413	1.04247	1.508705	1.058497	2.991059	4.469695
F_6_	Best	15,547.54	4403.94	651.3584	2841.421	1009.47	0.055643	0
	Mean	18,151.53	5395.454	1500.041	6914.513	3839.607	930.2275	2.94E−06
	SD	1093.599	411.362	584.8083	2225.258	1408.859	957.1977	2.94E−05
F_7_	Best	511.8067	112.6812	15.96008	110.5245	210.6031	51.73782	0
	Mean	587.6085	155.6537	21.72945	211.2711	265.203	131.404	3.64E−07
	SD	25.18078	15.83632	2.781115	42.39096	18.5789	38.00434	3.63E−06
F_8_	Best	10.05457	18.11648	2.599891	0.599873	1.399914	0.599873	0
	Mean	12.34569	21.19853	3.134814	0.830873	2.029959	1.104873	0.009265
	SD	0.966472	0.822021	0.27839	0.099184	0.226778	0.190361	0.081186
F_9_	Best	8.49E−07	5.35E−11	1.08E−09	0	1.43E−12	0	0
	Mean	2.98E−03	2.54E−05	1.72E−05	9.25E−09	1.67E−08	0	0
	SD	7.70E−03	7.88E−05	3.90E−05	1.75E−08	3.39E−08	0	0
F_10_	Best	77.50366	71.56383	14.6766	0.460693	3.379566	0.450844	0
	Mean	91.58769	83.56584	17.55798	1.319453	7.234517	1.909486	0.503791
	SD	2.434241	4.747136	1.236382	0.505237	1.954402	0.958307	4.897183
F_11_	Best	56.83658	34.3526	3.065453	0.088745	0.023951	0.000271	0
	Mean	68.01628	38.76511	3.687095	0.148389	0.111745	0.074045	1.10E−06
	SD	3.374358	1.686266	0.247853	0.023546	0.058802	0.114694	1.10E−05
F_12_	Best	333,012.9	284.4142	3805.585	0.424549	53.86077	0.013213	0
	Mean	474,307.6	471.6474	5370.455	0.904045	113.9655	2.278516	0.259769
	SD	44,714.95	90.6102	635.0582	0.30938	29.47293	5.882736	2.597688
F_13_	Best	1.05E + 09	5.38E + 08	8,042,041	0.188584	3122.341	0.018164	0
	Mean	1.42E + 09	6.99E + 08	12,104,315	0.501756	18,977.06	340.8279	0.001432
	SD	1.12E + 08	58,432,546	1,604,566	0.160563	8842.065	457.0865	0.01432
F_14_	Best	71,819.64	15.43768	954.7067	0.132864	10.13445	12.38795	46.96118
	Mean	97,864.79	19.82098	1262.973	0.269682	49.44706	22.25387	73.26776
	SD	11,197	1.451369	142.6338	0.075034	40.36055	4.672117	10.17666
F_15_	Best	70,339.89	16.00331	888.4887	0.130584	12.04421	16.55338	26.27337
	Mean	97,836.47	19.52131	1240.962	0.279098	54.91552	24.86934	72.60386
	SD	10,159.17	1.460158	139.9793	0.072831	56.50644	4.299523	11.2144
F_16_	Best	1872.414	0.527636	85.29447	0.008924	0.171064	3.42E−12	0
	Mean	2199.699	0.668883	108.5625	0.013944	0.89789	2.23E−09	0
	SD	134.2236	0.058032	10.19812	0.002377	0.567639	1.20E−08	0
F_17_	Best	223,877	130,660	8707	36	173	166	22
	Mean	268,117.9	148,673.9	10,812.77	84.51	299.23	559.02	32.41
	SD	13,451.02	7049.15	969.7187	31.88759	65.20935	160.5713	4.878514
F_18_	Best	5,588,993	5,588,993	195,795.3	867.3468	3839.556	1924.694	262.1536
	Mean	6,702,584	6,702,584	267,328.7	1195.725	6439.726	5520.177	420.8609
	SD	337,357.7	337,357.7	25,817.66	140.2667	1114.555	2013.876	839.9876
F_19_	Best	0.857868	0.47082	0.083849	0.504413	0.401207	0.318924	0
	Mean	0.892505	0.541511	0.106005	0.580524	0.494181	0.5283	0.002477
	SD	0.009596	0.024226	0.008691	0.031767	0.027936	0.047698	0.023212
F_20_	Best	0.000205	2.35E−05	2.79E−06	1.99E−05	1.28E−06	7.16E−06	9.65E−06
	Mean	0.018252	1.44E−03	1.85E−03	2.03E−03	1.73E−03	1.15E−03	1.13E−03
	SD	0.021241	1.14E−03	2.03E−03	1.80E−03	1.51E−03	1.02E−03	1.37E−03

**Figure 5:**
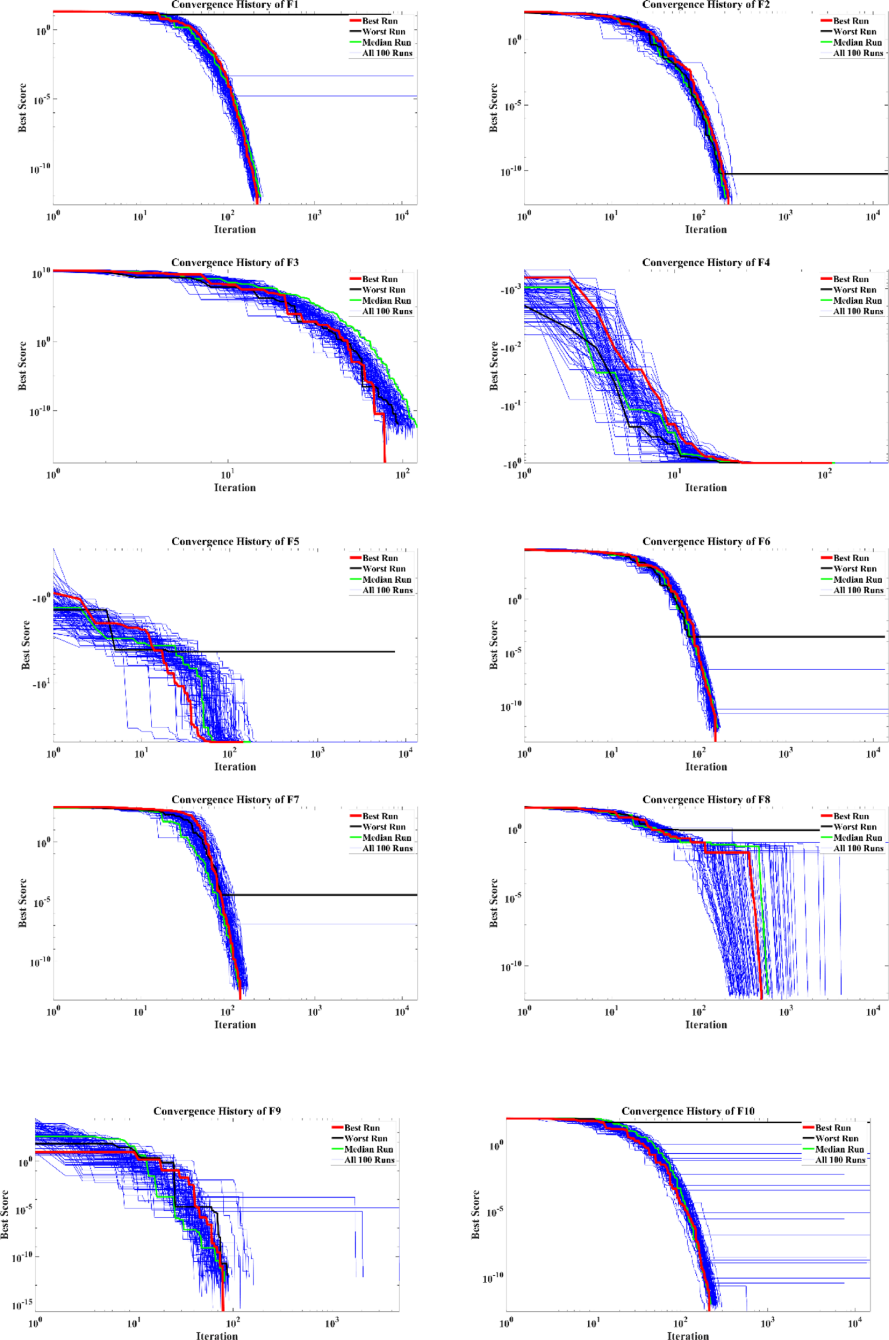

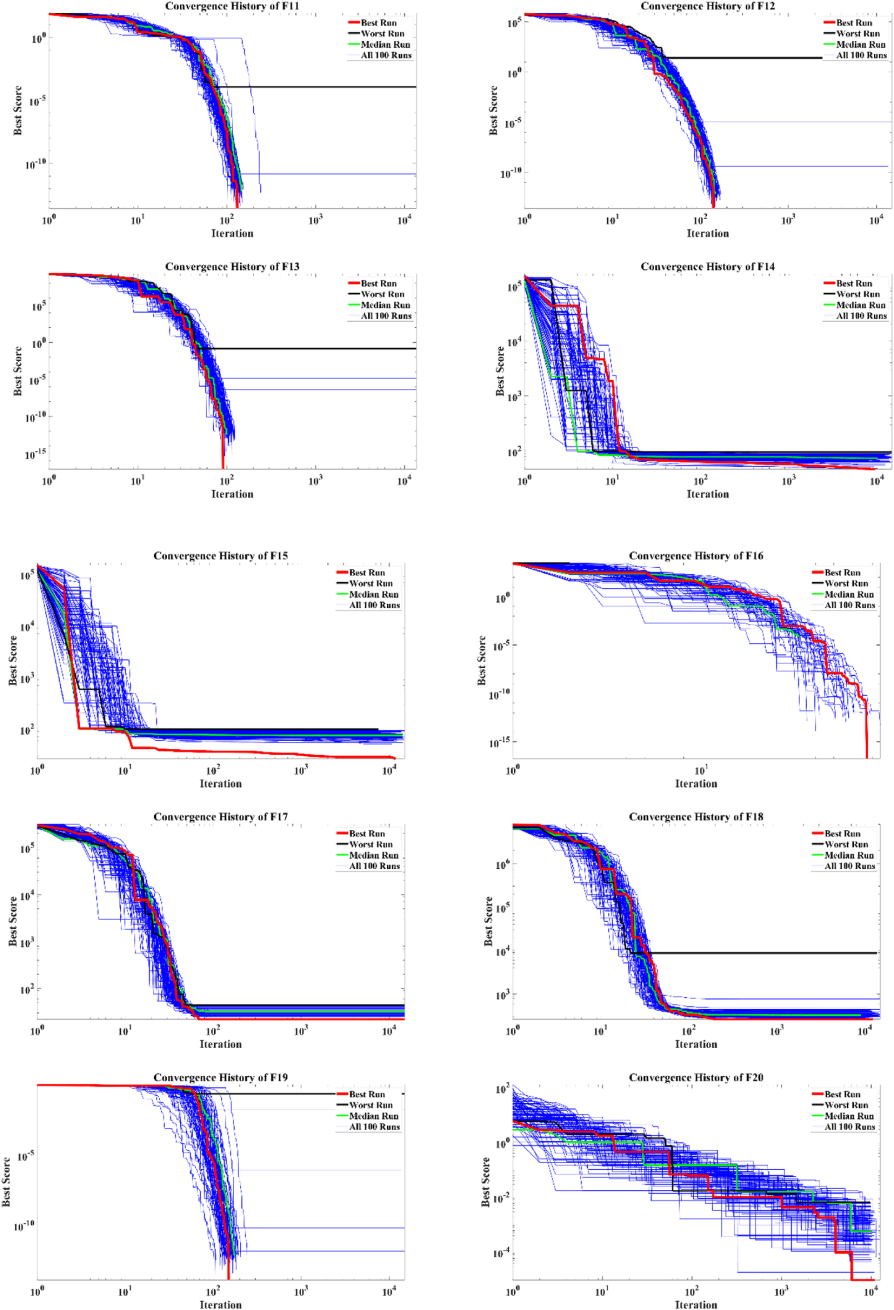
Convergence curves of the EVO for the mathematical functions.

The convergence curves of 100 optimization runs conducted by the EVO in dealing with the mathematical test functions are illustrated in Fig. 5. The best and worst runs, alongside the mean of all runs, are highlighted. By considering the convergence history of EVO, it can be concluded that the proposed algorithm is capable of performing fast optimization procedures in most of the considered problems, while the algorithm does not need to conduct predefined 150,000 objective function evaluations and is capable of reaching the tolerance of $1\times {10}^{-12}$ in a swift way.

## Statistical analysis

In this section, the results of the EVO and other alternative metaheuristic algorithms in dealing with the mathematical test functions are utilized for conducting a comprehensive statistical analysis. In this regard, four of the well-known statistical tests as the Kolmogorov Smirnov (KS) test for normality control, Wilcoxon (W) signed ranks test for comparing in a two-by-two procedure the summation and mean of the metaheuristics' ranks, and the Kruskal Wallis (KW) test for evaluating the overall rankings of different metaheuristic algorithms by comparing the mean of the metaheuristics' ranks. In Table 5, the results of the KS test are provided in which the p-value of this test is less than 0.05, so the hypothesis in the normal distribution of data is satisfied, and the non-parametric statistical tests can be utilized for further investigations.

**Table 5:** The KS test results (*p*-values) for different metaheuristic algorithms.

Main algorithm	Data type	Alternative metaheuristic algorithms					
		ACO	FA	HS	MVO	CSA	ISA
EVO	Best	8.42E−06	8.42E−06	8.42E−06	4.15E−05	8.42E−06	7.25E−04
	Mean	1.83E−04	1.83E−04	7.25E−04	2.32E−02	2.57E−03	2.32E−02
	SD	2.32E−02	5.91E−02	5.91E−02	1.35E−01	5.91E−02	4.97E−01
	OFE	8.42E−06	8.42E−06	8.42E−06	8.42E−06	8.42E−06	4.15E−05

**Figure 6:**
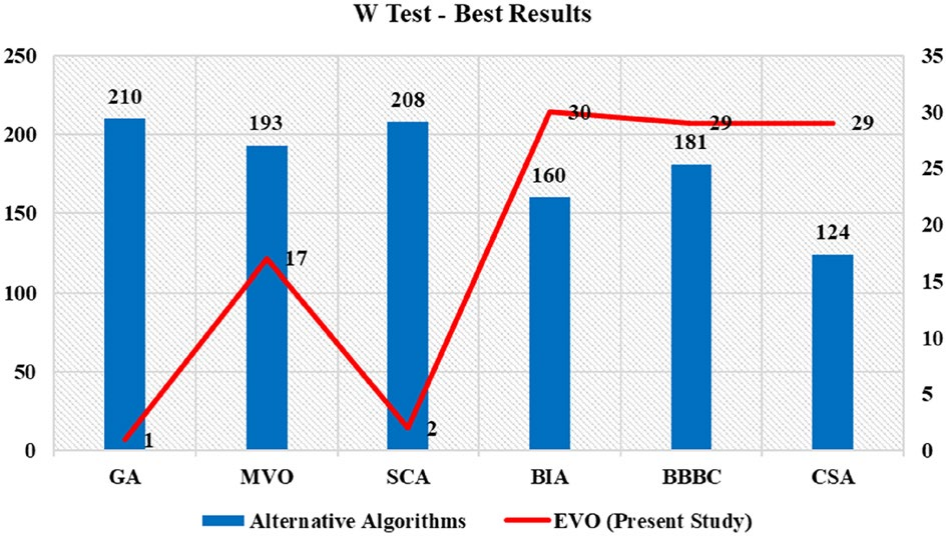
The W test results (mean of ranks) for different metaheuristic algorithms.

**Figure 7:**
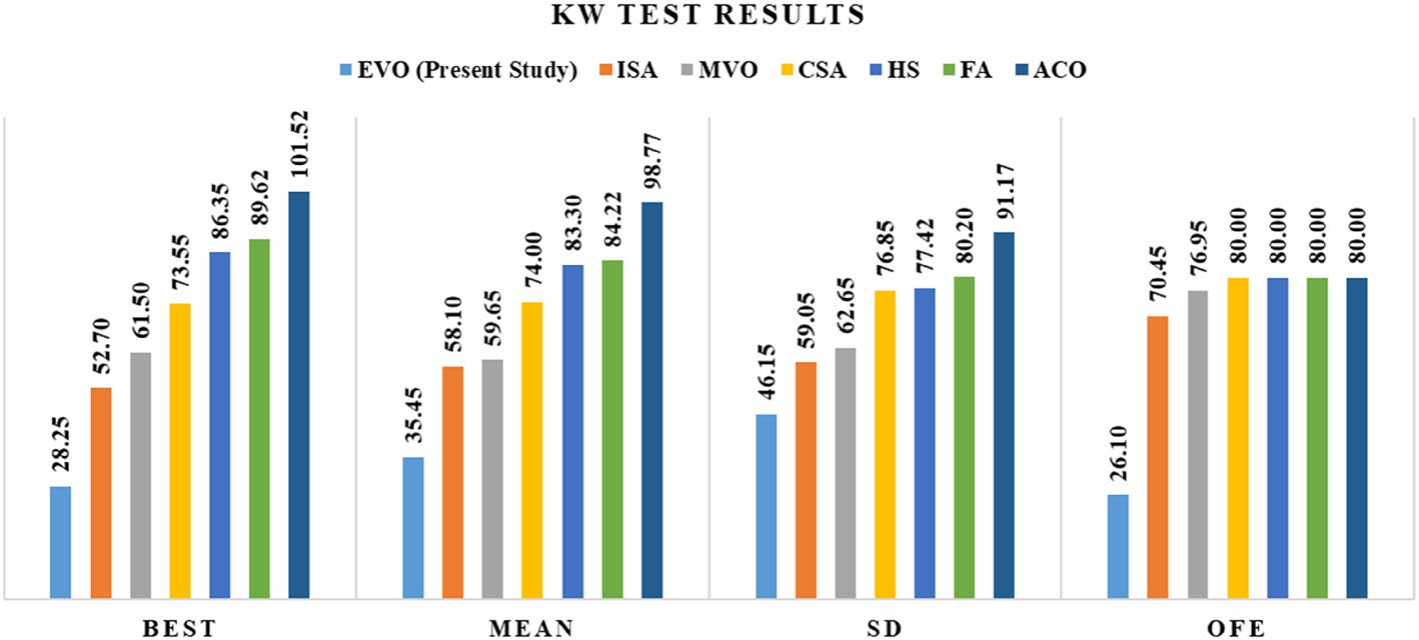
The KW test results including mean of the ranks for different metaheuristic algorithms.

In Fig. 6, the results of the W test are presented in which the mean ranks of different metaheuristic algorithms are provided and compared in a two-by-two manner regarding the best results, while the metaheuristics with a smaller mean of ranks are superior to the other algorithm. The EVO can provide better results with smaller means of ranks in most cases based on the results. In Fig. 7, the results of the KW statistical test, including the mean of ranks by considering all of the data sets, are presented in which the EVO is capable of outranking the other algorithms in all of the considered data sets.

## CEC 2020 complexity analysis & Big O notation

In most of the recently developed metaheuristic algorithms, the computational efforts of the algorithms in dealing with complex optimization problems have been of significant concern due to the increasing interest of artificial intelligence experts to provide computationally efficient algorithms for optimization purposes. In this regard, the computational time procedure of the CEC 2020 benchmark suit on bound constrained^60^ is utilized in which T_0_ represents the run time of a predefined mathematical process, T_1_ is the computational time of G1 function by considering 200,000 objective function evaluations, T_2_ is the computational time of the proposed algorithm (EVO) for 200,000 objective function evaluations of G1 function, and ${\widehat{\mathrm{T}}}_{2}$ is the mean of five individual T_2_. In Table 6, the computational time of the proposed EVO algorithm and other approaches are provided in which the capability of the EVO in competing with other algorithms is in perspective.

**Table 6:** The computational time of different algorithms in dealing with the CEC 2020 procedure.

Metaheuristics	Results (sec)			
	T_0_	T_1_	${\widehat{\mathrm{T}}}_{2}$	$({\widehat{\mathrm{T}}}_{2}-{\mathrm{T}}_{1})/{\mathrm{T}}_{0}$
IMODE^63^	0.01117	0.2235	0.3330	0.9780
j2020^64^	0 *	0.0465	0.1818	Inf **
GSK^65^	0.0411	1.12E−05	1.76E−05	1.58E−04
EVO (Present Study)	0.0186	0.0163	3.2271	172.6236

*It is a rounded value.

** unitless.

One of the well-known procedures for evaluating the computational complexity of the algorithms is the “Big O notation” “which is frequently used in computer science and is adopted in this paper for further investigations on EVO algorithm. By considering NP and D as the total number of initial solution candidates and the dimension of the optimization problem, respectively, the computational complexity of generating position vectors and calculating objective function are as O(NP × D) and O(NP) × O(F(x)) regarding the fact that F(x) represents the objective function of the optimization problem. In the main loop of the EVO, each line has a computational complexity of MxItr as the total number of iterations. The position updating process for each of the solution candidates in this loop can be conducted in two phases as if ${\mathrm{NEL}}_{\mathrm{i}}>\mathrm{EB}$, two new position vectors are created in one of the ${\mathrm{SL}}_{\mathrm{i}}>\mathrm{SB}$ and ${\mathrm{SL}}_{\mathrm{i}}\le \mathrm{SB}$ subphases so the computational complexity of O(MxItr × NP × D × 2) is determined in this phase. Regarding ${\mathrm{NEL}}_{\mathrm{i}}\le \mathrm{EB}$, only one new position vector is generated, so the complexity of O(MxItr × NP × D) is concerned in this case. For objective function evaluation in these two phases, the complexity of O(MaxIter × NP × D × 3) × O(F(x)) and O(MaxIter × NP × D) × O(F(x)) are determined, respectively.

## CEC 2020 real-world constrained optimization

**Figure 8:**
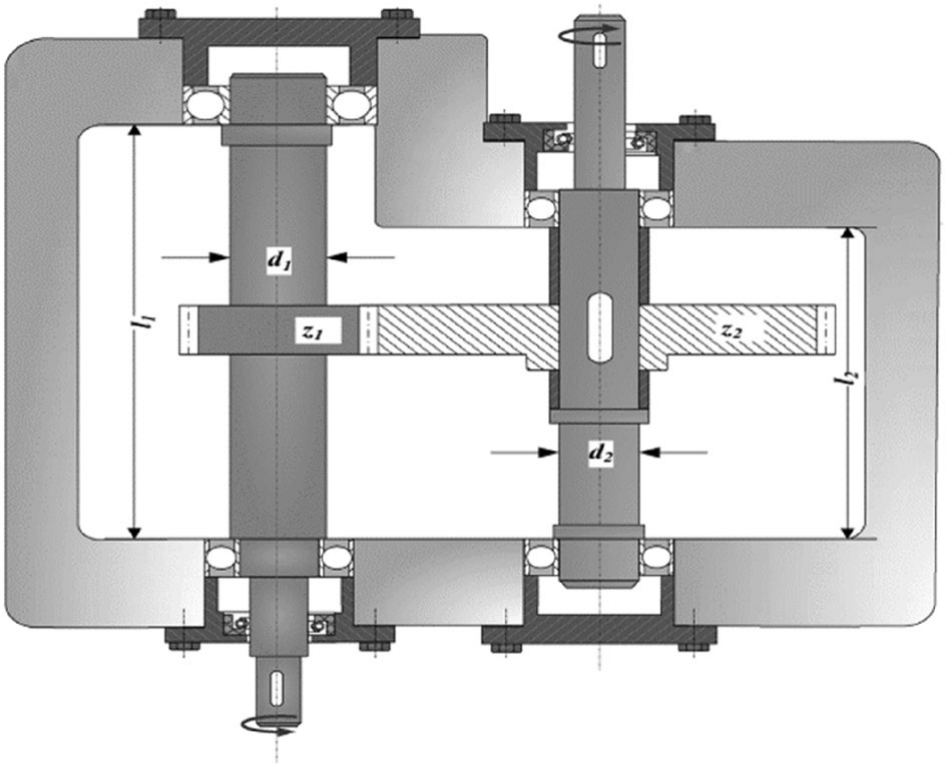
Schematic view of the speed reducer problem^66^.

**Figure 9:**
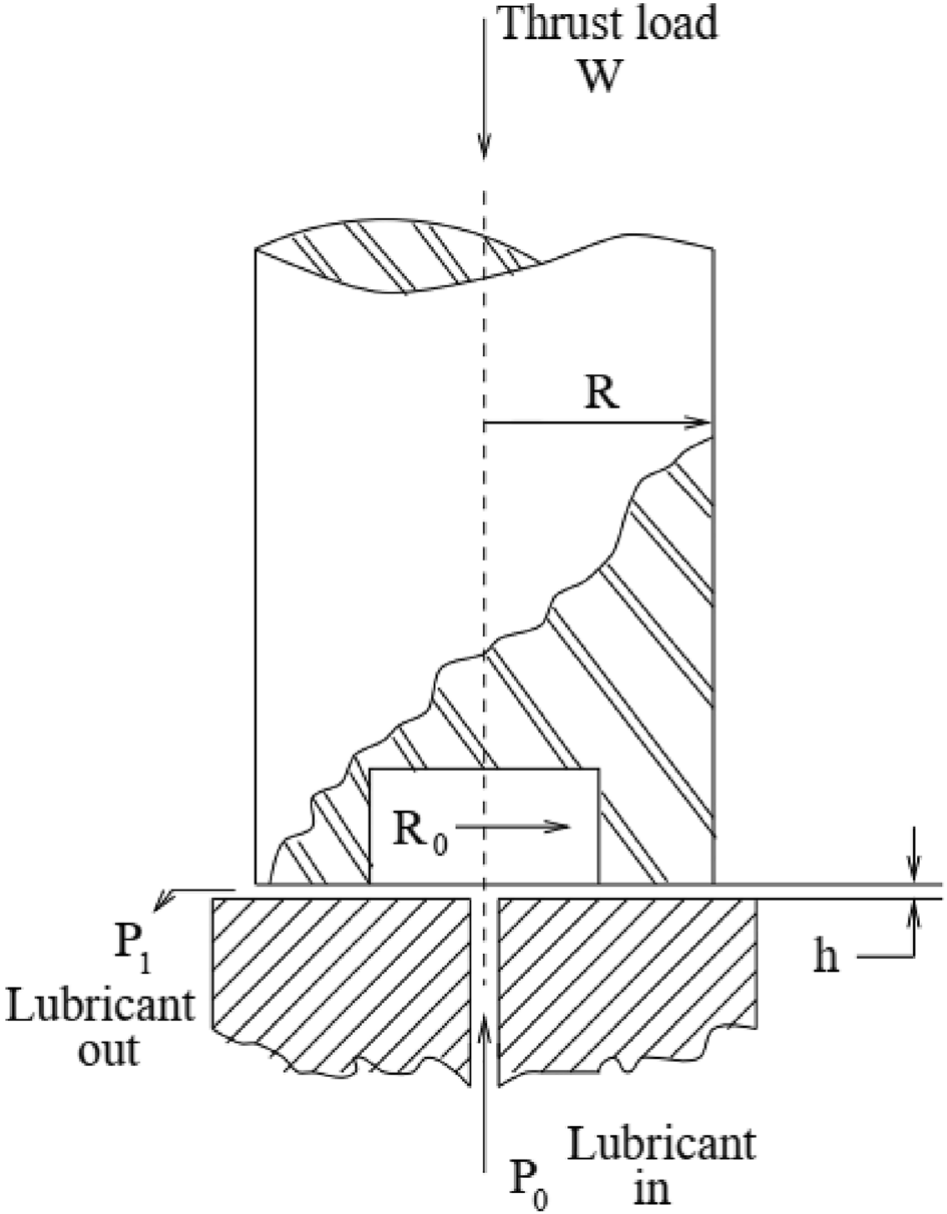
Schematic view of the hydro-static thrust bearing^66^.

**Figure 10:**
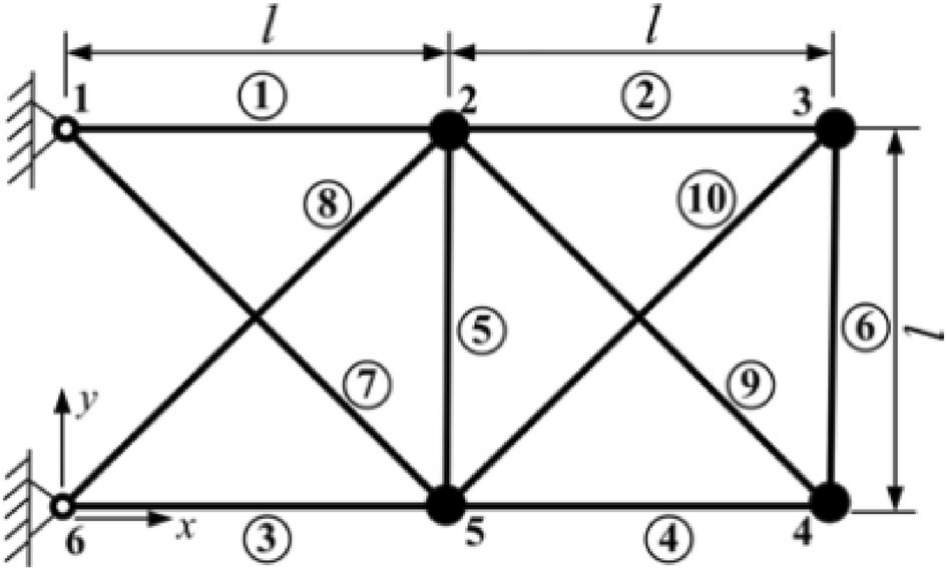
Schematic view of the ten-bar truss problem^66^.

**Figure 11:**
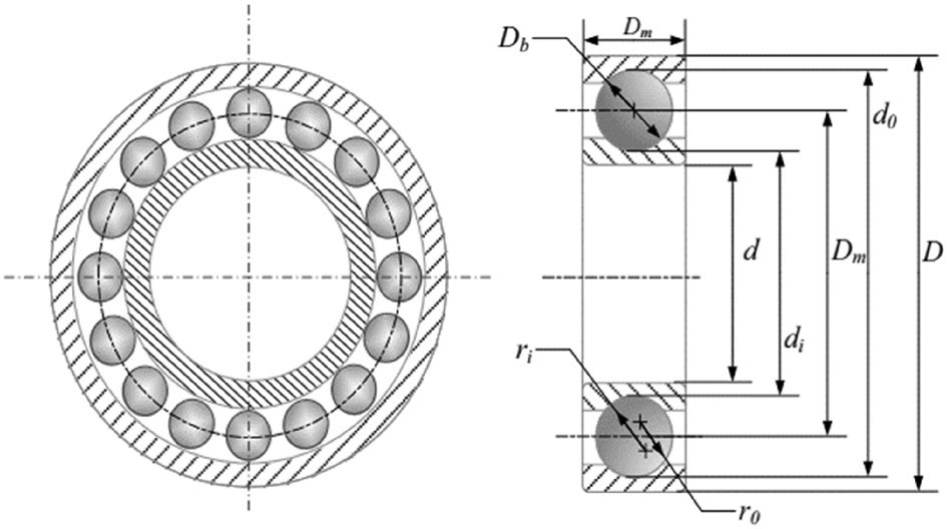
Schematic view of the rolling element bearing problem^66^.

Most of the time, the capability of the metaheuristic algorithms is considered through real-world optimization problems in which some sort of design constraints alongside the bound constraints should be handled for having feasible solutions. For this purpose, the real-world constraint optimization problems of CEC 2020^61^ are utilized in this paper. In Table 7, a summary of these engineering design problems is presented, while the complete mathematical formulations can be found in the literature. The schematic presentation of these problems is also presented in Figs. 8, 9, 10, 11. A total number of 30 independent optimization runs have been conducted using 20,000 function evaluations for statistical purposes. For constraint handling purposes, however, the well-known penalty approach with static co-efficient is utilized in the current study.

**Table 7:** Real-world constrained optimization problems.

No. (CEC No.)	Name	D	g	h
H_1_ (RC15)	Speed Reducer	7	11	0
H_2_ (RC25)	Hydro-Static Thrust Bearing	4	7	0
H_3_ (RC27)	Ten-Bar Truss	10	3	0
H_4_ (RC28)	Rolling Element Bearing	10	9	0

D, Dimensions; g, Number of inequality constraints; h, Number of equality constraints.

In Tables 8 and 9, the best and statistical results of EVO and other algorithms in dealing with the speed reducer problem are presented in which the optimum design variables alongside the design constraints are provided. Based on the results of best optimization runs conducted by different methods, EVO can provide 2994.42, which is the best among other approaches. Besides, EVO provides the means and worst of 2994.44 and 2994.46, respectively, which are better than other methods’ results.

**Table 8:** Best results of different approaches for the speed reducer problem.

	ES^67^	SBS^68^	CSA^69^	DE^70^	MGA^16^	CGO^66^	Present Study (EVO)
Best	3025.0050000000	3008.0800000000	3000.9810000000	2994.4710660000	2994.4436490000	2994.4388690000	2994.4259994948
b	3.5061630000	3.5061220000	3.5015000000	3.5000000000	3.5000066840	3.5000079560	3.5000002974
m	0.7008310000	0.7000060000	0.7000000000	0.7000000000	0.7000000000	0.7000006560	0.7000000004
z	17.0000000000	17.0000000000	17.0000000000	17.0000000000	17.0000000000	17.0000008100	17.0000005194
l_1_	7.4601810000	7.5491260000	7.6050000000	7.3000000000	7.3000000000	7.3005419270	7.3000041520
l_2_	7.9621430000	7.8593300000	7.8181000000	7.7153199100	7.7153272500	7.7153576930	7.7153347121
d_1_	3.3629000000	3.3655760000	3.3520000000	3.3502146700	3.3505953620	3.3505423910	3.3505420375
d_2_	5.3090000000	5.2897730000	5.2875000000	5.2866544700	5.2866584470	5.2866579300	5.2866555457
g_1_(x)	−0.0777000000	−0.0755000000	−0.0743000000	− 0.0739152	−2.1550556750	−2.1551222770	−2.1550034019
g_2_(x)	−0.2013000000	−0.1994000000	−0.1983000000	− 0.1979985	−98.1359464800	−98.1371022200	−98.1350729737
g_3_(x)	−0.4741000000	−0.4562000000	−0.4349000000	− 0.9999967	−1.9253722120	−1.9242737610	−1.9251203297
g_4_(x)	−0.8971000000	−0.8994000000	−0.9008000000	− 0.9999995	−18.3099259200	−18.3096983400	−18.3098233913
g_5_(x)	−0.0110000000	−0.0132000000	−0.0011000000	−0.6668526000	−0.0535900310	−0.0004371520	−0.0010647971
g_6_(x)	−0.0125000000	−0.0017000000	−0.0004000000	− 0.0000000	−0.0019196020	−0.0016664740	−0.0005189180
g_7_(x)	−0.7022000000	−0.7025000000	−0.7025000000	−0.7025000000	−28.1000000000	−28.0999882900	−28.0999996296
g_8_(x)	−0.0006000000	−0.0017000000	−0.0004000000	− 0.0000000	−0.0000095500	−0.0000066800	−0.0000004220
g_9_(x)	−0.5831000000	−0.5826000000	−0.5832000000	− 0.5833333	−6.9999904520	−6.9999933180	−6.9999995780
g_10_(x)	−0.0691000000	−0.0796000000	−0.0890000000	− 0.0513257	−0.3741069580	−0.3747283410	−0.3741910957
g_11_(x)	−0.0279000000	−0.0179000000	−0.0130000000	− 0.0000000	−0.0000029600	−0.0000340000	−0.0000136118

teeth module (m), face width (b), length of the first shaft between bearings (l1), the diameter of the first shaft (d1), number of teeth on pinion (z), length of the second shaft between bearings (l2), the diameter of the second shaft (d2).

ES, Evolution strategy; SBS, Socio-behavioural simulation; CSA, Cuckoo search algorithm;

DE, Differential evolution.

**Table 9:** Statistical results for the speed reducer problem considering different approaches.

Approaches	Best	Mean	Worst	Std-Dev
ES^67^	3025.0050000000	3088.7778000000	3078.5918000000	NA
SBS^68^	3008.0800000000	3012.1200000000	3028.2800000000	NA
CSA^69^	3000.9810000000	3007.1997000000	3.0090000000	4.9634000000
DE^70^	2994.4710660000	2994.4710660000	2994.4710660000	0.0000000000
MGA^16^	2994.4388690000	2994.4706500000	2996.5582370000	4.72E−16
CGO^66^	2994.4436490000	2994.4653970000	2995.5049330000	0.1102820000
Present Study (EVO)	2994.4259994948	2994.4438541726	2994.4633295494	0.0115141369

The best and statistical results of different algorithms, including the proposed EVO in dealing with the hydro-static thrust bearing design problem, are provided in Tables 10 and 11, respectively, including the optimum design variables and the design constraints. In dealing with the problem, EVO can provide the problem, EVO can provide 1619.55, which is the best among other approaches, while the best so far found in other approaches is for CGO, which calculated 1621.24. Meanwhile, EVO provides the means and worst of 1730.09 and 1899.34, respectively, demonstrating some superiority compared to other approaches.

**Table 10:** Comparison of the best solutions for the hydro-static thrust bearing design problem.

	COM^71^	CGS^72^	EA^73^	TLBO^38^	MGA^16^	CGO^66^	Present Study (EVO)
Best	2288.2268000000	2161.4215000000	1950.2860000000	1625.4427600000	1623.9809380000	1621.2461750000	1619.5525106188
R	7.1550000000	6.7780000000	6.2710000000	5.9557805026	5.9632415160	5.9634400230	5.9567022667
R_0_	6.6890000000	6.2340000000	12.9010000000	5.3890130519	5.3959079890	5.3955878610	5.3896315105
µ	0.0000083210	6.096 E−06	0.0000056050	0.0000053586	0.0000053800	0.0000053600	0.0000053812
Q	9.1680000000	3.8090000000	2.9380000000	2.2696559728	2.2822425050	2.2648221880	2.2751206437
g_1_(x)	−11,086.7430000000	−8329.7681000000	−2126.8673400000	−0.0001374735	−144.9586796000	−9.0788651780	−10.5323413309
g_2_(x)	−402.4493000000	−177.3527000000	−68.0396000000	−0.0000010103	−1.1948020210	−2.5136231960	−0.2665016758
g_3_(x)	−35.0571960000	−10.6845430000	−3.7051910000	−0.0000000210	−0.3724500270	−0.0021106440	−0.3581746345
g_4_(x)	−0.0015420000	−0.0006520000	−0.0005590000	−0.0003243625	−0.0003291500	−0.0003248340	−0.0003275759
g_5_(x)	−0.4660000000	−0.5440000000	−0.6660000000	−0.5667674507	−0.5673335270	−0.5678521610	−0.5670707561
g_6_(x)	−0.0001440000	−0.0007170000	−0.0008050000	−0.0009963614	−0.0009963550	−0.0009963660	−0.0009963610
g_7_(x)	−563.6444010000	−83.6182210000	−849.7186830000	−0.0000090762	−4.1442588760	−15.3591184600	−2.8341205598

bearing step radius (R), recess radius (R0), oil viscosity (µ), flow rate (Q).

*COM* Classic optimization method, *CGS* Combined genetic search, *EA* Evolutionary algorithm, *TLBO* Teaching–learning-based optimization.

**Table 11:** Statistical results of different approaches for the hydro-static thrust bearing design problem.

Approaches	Best	Mean	Worst	Std-Dev
EGWO^74^	1625.4646700000	1627.7441980000	1650.6987470000	3.8155469730
JA^75^	1625.4427100000	1796.8936700000	2104.3776000000	0.2100000000
TLBO^38^	1625.4427600000	1797.7079800000	2096.8012000000	0.1900000000
MGA^16^	1621.2461750000	1739.1567290000	1992.9613050000	0.1100000000
CGO^66^	1621.2461750000	1706.0414310000	1981.1732950000	64.4989571200
Present Study (EVO)	1619.5525106188	1730.0997954158	1899.3444719503	72.9271039971

EGWO, Enhanced grey wolf optimizer; JA, Jaya algorithm.

Tables 12 and 13 provide the best and statistical results of different algorithms, including the proposed EVO, in dealing with the ten-bar truss design problem. It can be concluded that the proposed EVO can provide 524.92, which is much better than the previously reported results of 529 and 530. In addition, EVO provides better means, and worst values demonstrate this novel algorithm’s superiority compared to other approaches.

**Table 12:** Comparison of the best solutions for the ten-bar truss design problem.

	UPTR^76^	SLP^77^	TLBO^78^	ECSS^79^	MGA^16^	CGO^66^	Present study (EVO)
Best	544.7000000000	534.5700000000	530.7600000000	529.2500000000	529.1204229000	526.0057179000	524.9297706988
A_1_	36.3800000000	35.1480000000	35.4940000000	39.5690000000	36.7641600000	35.9271100000	0.0035129553
A_2_	12.9410000000	13.1690000000	14.7770000000	16.7400000000	16.2989700000	13.1573200000	0.0014623757
A_3_	35.7640000000	37.6900000000	36.2030000000	34.3610000000	37.9437800000	34.4322800000	0.0034910422
A_4_	18.3140000000	19.5560000000	15.3870000000	12.9940000000	16.5108700000	15.5951500000	0.0014785733
A_5_	3.0020000000	1.0870000000	0.6451000000	0.6450000000	0.6590000000	0.6580000000	0.0000646513
A_6_	5.4330000000	4.8440000000	4.5896000000	4.8020000000	4.5748900000	4.6283500000	0.0004607633
A_7_	20.9890000000	18.3140000000	23.2110000000	26.1820000000	22.9402300000	21.3776000000	0.0023593717
A_8_	24.1400000000	27.4150000000	24.5610000000	21.2600000000	22.6318500000	27.0243000000	0.0023952278
A_9_	9.7530000000	12.5620000000	12.4820000000	11.7660000000	10.8789200000	13.6223300000	0.0012999784
A_10_	18.1020000000	12.1060000000	12.3240000000	11.3920000000	11.5364300000	11.0003500000	0.0011962322

cross-sectional areas of the truss bars (A1, A2, A3, A4, A5, A6, A7, A8, A9, A10).

IPTR, Interior Point Trust Regio; SLP, Sequential Linear Programming; ECSS, Enhanced Charged System Search.

**Table 13:** Statistical results of the MGA method for the ten-bar truss bearing design problem.

Approaches	Best	Mean	Worst	Std-Dev
MGA^16^	529.1204229000	534.6843574000	548.0179132000	26.3365167500
CGO^66^	526.0057179000	531.3864551793	534.1262390000	2.8915658990
Present Study (EVO)	524.9297706988	528.3155150968	532.0617359800	1.9129280976

Tables 14 and 15 present the best and statistical results of EVO and other algorithms in dealing with the rolling element bearing design problem (as a maximization problem), in which the optimum design variables and the design constraints are provided. Based on the best optimization runs conducted by different methods, EVO can provide 81,859.74, while the ALO with 85,546.63 is the best algorithm in providing the best result in this case. However, the EVO is capable of competing with the ALO and other approaches by providing mean and worst values of 81,110.32 and 80,212.09, respectively.

**Table 14:** Comparison of the best solutions for the rolling element bearing design problem.

	TLBO^38^	ABC^80^	GWO^80^	ALO^80^	MGA^16^	CGO^66^	Present Study (EVO)
Best	81,859.7400000000	85,428.2495000000	85,529.0830000000	85,546.6377000000	83,912.8798300000	83,918.4925300000	81,859.7415974169
D_m_	21.4255900000	125.6599000000	125.7090000000	125.7180000000	125.0002787000	125.0000000000	125.7190556147
D_b_	125.7191000000	21.4086200000	21.4231600000	21.4252420000	21.8745119200	21.8750000000	21.4255902408
Z	11.0000000000	11.0000000000	11.0000000000	11.0000000000	10.7770658300	10.7770090500	10.6955328415
f_i_	0.5150000000	0.5150000000	0.5150000000	0.5150000000	0.5150008220	0.5150000000	0.5150000000
f_0_	0.5150000000	0.5150000000	0.5293220000	0.5157018000	0.5150029930	0.5150000000	0.5150000000
K_Dmin_	0.4242660000	0.4271660000	0.4208670000	0.4541646000	0.4059083530	0.4000000000	0.4631829367
K_Dmax_	0.6339480000	0.6688490000	0.6332960000	0.6464928000	0.6555880200	0.6462005260	0.6999265065
ε	0.3000000000	0.3000000000	0.3002240000	0.3000122000	0.3000041550	0.3000000000	0.3000000000
e	0.0688580000	0.0713860000	0.0200000000	0.0638003000	0.0775449260	0.0501524450	0.0634315198
ζ	0.7994980000	0.6000000000	0.6194320000	0.6107592000	0.6000000000	0.6000000000	0.6042131085

pitch diameter (Dm), ball diameter (Db), total number of balls (Z), inner raceway curvature coefficient (fi), and the outer raceway curvature coefficient (f0).

*ABC* Artificial bee colony, *GWO* Grey wolf optimizer, *ALO* Ant lion optimizer.

**Table 15:** Statistical results of different approaches for the rolling element bearing design problem.

Approaches	Best	Mean	Worst	Std-Dev
TLBO^38^	81,859.7400000000	81,438.9870000000	80,807.8551000000	0.6600000000
ABC^80^	85,428.2495000000	85,121.7544000000	83,859.0851000000	362.5700000000
GWO^80^	85,529.0830000000	83,395.0849000000	43,543.4508000000	8224.5000000000
ALO^80^	85,546.6377000000	84,032.8636000000	73,872.8164000000	3121.8000000000
MGA^16^	83,912.8798300000	83,892.2564700000	83,711.2131700000	23.6584100000
CGO^66^	83,918.4925300000	83,916.5974900000	83,829.8000000000	10.5358000000
Present Study (EVO)	81,859.7415974169	81,110.3219736893	80,212.0966120356	522.1000790005

## Conclusions

Energy Valley Optimizer (EVO) is proposed as a novel metaheuristic algorithm inspired by the advanced principles of physics regarding stability and different modes of decay in particles. For evaluation purposes, 20 unconstrained mathematical test functions with 100 independent optimization runs and the maximum number of 150,000 objective function evaluations alongside the latest Competitions on Evolutionary Computation (CEC), including the CEC 2020 on real-world optimization are utilized. The key results and main findings of this research paper are summarised as follows:

- In dealing with the unconstrained mathematical test functions, EVO can outrank the other alternative metaheuristic algorithm and converge to the global best solutions in most cases.
- EVO can converge to the global best solution with the lowest objective function evaluations, demonstrating this novel algorithm’s efficiency from a computational point of view.
- Based on the W statistical test results, the EVO can provide better results with smaller means of ranks compared to other algorithms in a two-by-two manner.
- The results of the KW statistical test, including the mean of ranks, demonstrate that the EVO can out-ranked the other algorithms in all of the considered data sets.
- Considering the constrained design examples of the CEC 2020 on real-world problems, the EVO can reach better solutions than other algorithms from the literature.
- Based on the best optimization runs conducted by different methods in dealing with the speed reducer problem, EVO can provide 2994.42, which is the best among other approaches.
- EVO can provide 1619.55 for the hydro-static thrust bearing design problem, which is the best among other approaches, while the best so far found in other approaches is for CGO, which calculated 1621.24.
- The proposed EVO can provide 524.92 for the ten-bar truss design problem, which is much better than the previously reported results of 529 and 530.
- Regarding the rolling element bearing design problem, EVO can provide 81,859.74, as the best optimum solution alongside mean and worst values of 81,110.32 and 80,212.09, respectively.

Based on the results and conducted analysis, the main reason for the superiority of the EVO algorithm compared to other mentioned metaheuristics algorithms is threefold: parameter-free, fast convergence behavior, and the lowest possible objective function evaluation. The proposed EVO should be tested for future studies utilizing complex optimization problems in different fields, including real-size engineering design problems.

## Data Availability

The datasets used and/or analysed during the current study available from the corresponding author on reasonable request.

## Abbreviations

GA: Genetic algorithm
CEC: Competitions on evolutionary computation
DE: Differential evolution
N: Number of neutrons
PSO: Particle swarm optimisation
Z: Number of protons
FA: Firefly algorithm
α: Dense and positively charged particles
ACO: Ant colony optimization
β: Negatively charged particles
HS: Harmony search
γ: Photons with higher levels of energy
BBBC: Big-bang big-crunch
$\mathrm{n}$: Total number of particles
MVO: Multiverse algorithm
$\mathrm{d}$: Dimension of the considered problem
CGO: Chaos game optimization
${\mathrm{x}}_{\mathrm{i}}^{\mathrm{j}}$: The jth decision variable for determining the initial position of the ith candidate
PO: Projectiles optimisation
${\mathrm{x}}_{\mathrm{i},\mathrm{min}}^{\mathrm{j}}$: Lower bounds of the jth variable in the ith candidate
ALO: Ant lion optimizer
${\mathrm{x}}_{\mathrm{i},\mathrm{max}}^{\mathrm{j}}$: Upper bounds of the jth variable in the ith candidate
AISA: Adolescent identity search algorithm
EB: Enrichment bound
MGA: Material generation algorithm
NEL: Neutron enrichment level
AOS: Atomic orbital search
${\mathrm{NEL}}^{\mathrm{i}}$: Neutron enrichment level of the ith particle
AHO: Archerfish hunting optimizer
${\mathrm{SL}}^{\mathrm{i}}$: Stability level of the ith particle
ICA: Imperialistic competitive algorithm
$\mathrm{BS}$: Particles with best stability levels
WOA: Whale optimization algorithm
$\mathrm{WS}$: Particles with worst stability levels
HHO: Harris hawks optimization
${\mathrm{X}}_{\mathrm{i}}^{\mathrm{New}1}$: Newly generated particle in the universe
AOA: Arithmetic optimization algorithm
${\mathrm{X}}_{\mathrm{i}}$: Current position vector of the ith particles (solution candidates) in the universe
EVO: Energy valley optimizer
${\mathrm{x}}_{\mathrm{i}}^{\mathrm{j}}$: The jth decision variable
CSA: Cuckoo search algorithm
${\mathrm{D}}_{\mathrm{i}}^{\mathrm{k}}$: Total distance between the ith particle and the kth neighbouring particle
IMODE: Improved multi-operator differential evolution
EBOwithCMAR: Effective butterfly optimizer with covariance matrix adapted retreat phase
${\mathrm{SL}}^{\mathrm{i}}$: Stability level of the ith particle
HSES: Hybrid sampling evolution strategy
LSHADE-cnEpSin: LSHADE with an ensemble sinsoidal parameter adaptation
LSHADE-SPACMA: LSHADE with semi-parameter adaptation and covariance matrix adaptation
${\mathrm{X}}_{\mathrm{CP}}$: Position vector for the centre of particles
j 2020: Developed differential evolution
GSK: Gaining sharing knowledge based algorithm
ES: Evolution strategy
SBS: Socio-behavioural simulation
COM: Classic optimization method
CGS: Combined genetic search
TLBO: Teaching–learning-based optimization
EGWO: Enhanced grey wolf optimizer
JA: Jaya algorithm
IPTR: Interior point trust region
SLP: Sequential linear programming
ECSS: Enhanced charged system search
ABC: Artificial bee colony
GWO: Grey wolf optimizer
${\mathrm{r}}_{1}$: Random numbers in the range of [0, 1]
${\mathrm{X}}_{\mathrm{Ng}}$: Position vector of the neighbouring particle around the ith particle
${\mathrm{r}}_{2}$: Random numbers in the range of [0, 1]
${\mathrm{r}}_{3}$: Random numbers in the range of [0, 1]
${\mathrm{r}}_{4}$: Random numbers in the range of [0, 1]
OFE: Objective function evaluations
SD: Standard deviation
KS: Kolmogorov Smirnov
W: Wilcoxon
KW: Kruskal Wallis
D: Dimension
g: Number of inequality constraints
h: Number of equality constraints
